# Irremediability in psychiatric euthanasia: examining the objective standard

**DOI:** 10.1017/S0033291722002951

**Published:** 2023-09

**Authors:** Marie E. Nicolini, EJ Jardas, Carlos A. Zarate, Chris Gastmans, Scott Y. H. Kim

**Affiliations:** 1 Department of Bioethics, National Institutes of Health, 10 Center Drive, Room 1C118, Bethesda, Maryland 20892, USA; 2 Center for Biomedical Ethics and Law, KU Leuven, Kapucijnenvoer 35 – Box 7001, 3000 Leuven, Belgium; 3 Section on the Neurobiology and Treatment of Mood Disorders, Experimental Therapeutics and Pathophysiology Branch, National Institutes of Mental Health, 6001 Executive Boulevard, Room 6200, MSC 9663, Bethesda, MD 20892, USA

**Keywords:** aid-in-dying, assisted suicide, euthanasia, irremediability, precision psychiatry, psychiatry, treatment-resistant depression

## Abstract

**Background:**

Irremediability is a key requirement for euthanasia and assisted suicide for psychiatric disorders (psychiatric EAS). Countries like the Netherlands and Belgium ask clinicians to assess irremediability in light of the patient's diagnosis and prognosis and ‘according to current medical understanding’. Clarifying the relevance of a default objective standard for irremediability when applied to psychiatric EAS is crucial for solid policymaking. Yet so far, a thorough examination of this standard is lacking.

**Methods:**

Using treatment-resistant depression (TRD) as a test case, through a scoping review in PubMed, we analyzed the state-of-the-art evidence for whether clinicians can accurately predict individual long-term outcome and single out irremediable cases, by examining the following questions: (1) What is the definition of TRD; (2) What are group-level long-term outcomes of TRD; and (3) Can clinicians make accurate individual outcome predictions in TRD?

**Results:**

A uniform definition of TRD is lacking, with over 150 existing definitions, mostly focused on psychopharmacological research. Available yet limited studies about long-term outcomes indicate that a majority of patients with long-term TRD show significant improvement over time. Finally, evidence about individual predictions in TRD using precision medicine is growing, but methodological shortcomings and varying predictive accuracies pose important challenges for its implementation in clinical practice.

**Conclusion:**

Our findings support the claim that, as per available evidence, clinicians cannot accurately predict long-term chances of recovery in a particular patient with TRD. This means that the objective standard for irremediability cannot be met, with implications for policy and practice of psychiatric EAS.

## Introduction

A few countries in the world permit euthanasia and/or assisted suicide based primarily on a psychiatric disorder (psychiatric EAS), including Belgium, the Netherlands, Luxembourg, Switzerland, and Canada as of March 2023 (CCA, 2018; Griffith, Weyers, & Adams, 2008; Rukavina, 2019). One of the key requirements for psychiatric EAS in the Netherlands and Belgium is irremediability, or the lack of reasonable treatment options (Box 1). For example, the Dutch law states that a physician must ‘come to the conclusion, together with the patient, that there is no reasonable alternative in the patient's situation’ (Dutch Act, 2002). Existing Dutch and Belgian guidelines for clinicians state that the requirement ‘must be assessed in light of the diagnosis and prognosis’ (Euthanasia Code, 2018), from an ‘objective medical-psychiatric perspective’ and ‘according to current medical understanding’ (NVVP, 2018; VVP et al., 2017). In contrast, the Canadian law explicitly relies on a subjective judgment of irremediability, where remediable is defined by what a patient considers acceptable (CCA, 2018).

Box 1. Background information

Psychiatric EAS in the Netherlands and Belgium

Legal requirements for EAS

According to the Dutch Termination of Life on Request and Assisted Suicide Act (2002), the substantive requirements are that the attending physician must: be satisfied that the patient's request is voluntary and well-considered; be satisfied that the patient's suffering is unbearable and without prospect of improvement; have come to the conclusion, together with the patient, that there is no reasonable alternative in the patient's situation; have consulted at least one other, independent physician and have exercised due medical care in terminating the patient's life (Euthanasia Code, 2018; Onwuteaka-Philipsen et al., 2017). According to the Belgian 2002 Act Concerning Euthanasia, the physician must: come to the conviction, together with the patient, that there is no reasonable alternative in his/her condition and the request is voluntary; ascertain the continued physical or mental suffering of the patient and consult another physician about the serious and incurable nature of the disorder. If the patient is not expected to die in the near future, the following requirements apply in the Belgian Act: a second physician, a psychiatrist or a specialist in the disorder in question, needs to be consulted, and there should be at least one month between the patient's written request and the performance of euthanasia (Jones, Gastmans, and MacKellar, 2017).

Process and oversight systems for EAS

The Belgian Act requires that the physician consult a second physician – a psychiatrist in cases of psychiatric EAS – and requires a waiting time of at least one month for all non-terminally ill cases. While the Dutch law requires that the physician consults at least one other, independent physician, it does not specify that this be a psychiatrist for psychiatric EAS cases. However, in these cases, a psychiatric consultation is required by the Dutch Euthanasia Review Committees. Both countries have established services providing such consultants: Support and Consultation for Euthanasia in the Netherlands (SCEN) and Life End Information Forum (LEIF) in Belgium (Van Wesemael, Cohen, Onwuteaka-Philipsen, Bilsen, and Deliens, 2009). All EAS cases need to be reported post-hoc to the Regional Euthanasia Review Committees and the Federal Control and Evaluation Committee on Euthanasia, respectively in the Netherlands and Belgium. These committees review the EAS reports to assess whether the physician who performed EAS conformed to the legal due care criteria (Euthanasia Code, 2018; Jones, Gastmans, and MacKellar, 2017).

Evolving situation in Canada

The Canadian Medical Assistance in Dying (MAID) law enacted in 2016 stated that, to receive MAID, a person must be capable of making health decisions, have a grievous and irremediable medical condition, have made a voluntary request that was not the result of extremal pressure. To meet the ‘grievous and irremediability medical condition’ requirement, a person needs to: (a) have a serious and incurable illness, disease or disability; (b) be in an advance state of irreversible decline in capability, (c) the illness, the disease or disability or that state of decline causes them enduring physical or psychological suffering that is intolerable to them and that cannot be relieved under conditions that they consider acceptable; (d) their natural death has become reasonably foreseeable, taking into account all of their medical circumstances, without a prognosis necessarily having been made as to the specific length of time that they have remaining (CCA, 2018). Current Parliamentary discussions are ongoing, after a Quebec Superior Court stated the ‘reasonably foreseeable death’ requirement is unconstitutional, which led to the new Bill C7 (Bill C-7, 2021; Rukavina, 2019). As of March 2021, Canada has a commitment under its new law to legalize MAID based on a sole mental disorder (with a sunset provision of 2 years, going into effect March 2023) (Bryden, 2021). As of March 2022, a Parliamentary Review Committee is tasked with a comprehensive review of the provision of the Criminal Code relating to MAID and its applications, including MAID based on a mental disorder (Bill C-7, 2021; Gallant, 2022).

There is considerable conceptual debate about how irremediability should be defined in the context of psychiatric EAS, and whether an objective or subjective standard should prevail (Gaind, 2020; Nicolini, Kim, Churchill, & Gastmans, 2020a; Schuklenk, 2019; Sinyor & Schaffer, 2020; Smith, 2020; van Veen, Ruissen, & Widdershoven, 2020). The question of whether clinicians can, on an objective standard, accurately determine irremediability and prognosis in psychiatry is the single most contested claim in the professional debate about the practice (Nicolini et al., 2020a). Given pressing policy discussions about psychiatric EAS in Canada and elsewhere, clarifying whether the objective standard for irremediability is relevant is of crucial importance for solid policymaking and implementation of psychiatric EAS.

Discussions have repeatedly invoked ‘the person with treatment-resistant depression’ as the paradigm case of an irremediable psychiatric condition (Blikshavn, Husum, & Magelssen, 2017; Broome & de Cates, 2015; Miller, 2015; Schuklenk & van de Vathorst, 2015; Steinbock, 2017), often assuming that treatment-resistant depression (TRD) is, by definition, irremediable. Yet what does it mean for a clinician to assess prognosis and irremediability in a particular case, ‘according to current medical understanding’? Rooney et al., have rightly argued that assessing irremediability is to ‘perform a cost-benefit analysis of given treatments on a case-by-case basis, making medical decisions based on the statistically likely outcome’ (Rooney, Schuklenk, & van de Vathorst, 2018). For these medical decisions, they go on to argue, ‘evidence-based metrics for staging TRD, like the Maudsley Staging Method […] can be effective tools to help single out irremediable cases’. A thorough examination of whether clinicians can indeed single out irremediable cases in psychiatry –based on clinical judgment and/or on available tools– is lacking. This paper aims to address the glaring empirical gap in the debate over psychiatric EAS.

Focusing on TRD as a test case, we examine three claims relevant to the clinical assessment of prognosis and long-term outcome in a particular patient requesting psychiatric EAS, by asking in a stepwise approach: (1) What is the range of existing definitions of TRD? (2) What is known about the long-term outcomes of persons with TRD? and (3) What is the state-of-the-art regarding individual outcome prediction for a person with TRD? We then discuss how these findings inform the debate about irremediability in the context of psychiatric EAS.

## Methods

We reviewed the state-of-the-art evidence for the claim that clinicians can or cannot predict long-term chances of recovery in a patient with TRD through a scoping review, by asking the following three questions (Box 2): (1) Is there a uniform definition of TRD, i.e., a shared understanding of what clinicians mean by the term, (2) Can clinicians predict group-level long-term outcomes of TRD, i.e., what do we know about population-level long-term outcomes and their predictors, and (3) Can clinicians make accurate individual outcome predictions in a person with TRD, i.e., can they accurately determine who will and who will not achieve recovery in practice.

Box 2. Search strategy and selection criteria.

**Fig. 1.**
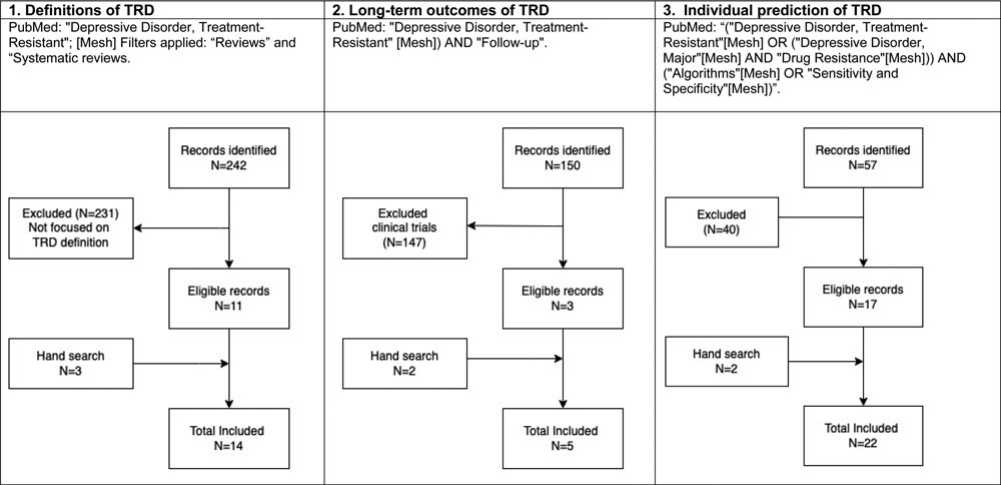
Search strategy and selection process.

We performed a scoping review focusing on three research questions, namely, what is the current state-of-the-art evidence about (1) definitions of TRD (2) long-term outcomes of TRD (3) individual prediction of TRD (Fig. 1). For the first research question about definitions of TRD, one author (M.N) performed a broad search in PubMed with no date restriction (Oct 6, 2020): (‘Depressive Disorder, Treatment-Resistant’ [Mesh]) yielded 1525 results. The question of how to define TRD has been extensively discussed in the literature. The aim was to examine the evidence for the (narrow) question of whether there is – or is not- a single definition of TRD. Hence, we further specified the search strategy to (systematic) reviews on the subject, by using PubMed filters ‘Reviews’ and ‘Systematic reviews’, yielding 242 results. Reviews focusing on definitions and concepts of TRD were included; reviews about specific or novel therapeutic strategies for TRD (pharmacology, psychotherapy, neuromodulation, basic research) were excluded. Reviews focusing on children and adolescents were excluded. 11 references were included, and another 3 included through hand search, for a total of 14 references.

For the second research question about long-term outcomes of TRD, M.N. used the following string: (‘Depressive Disorder, Treatment-Resistant’ [Mesh]) AND ‘Follow-up’), yielding 150 references. Inclusion criteria were publications focusing on (1) unipolar TRD, and (2) medium to long-term outcome at follow-up. The latter focused on naturalistic studies, excluding clinical trials where participants received adjunctive and/or experimental treatment. Medium to longer-term was defined as a period going beyond the usual period of several weeks or months or more as part of a clinical trial. Three publications were included, two additional references were yielded through hand search of the references, one of which was not indexed as ‘treatment-resistant’ as it was published before the specific MeSH term was introduced in PubMed in 2012.

For the third research question about individual prediction of TRD, one author (E.J.) performed a search with a broad and inclusive MeSH term and no date restriction: ‘(“Depressive Disorder, Treatment-Resistant”[Mesh] OR (“Depressive Disorder, Major”[Mesh] AND “Drug Resistance”[Mesh])) AND (“Algorithms”[Mesh] OR “Sensitivity and Specificity”[Mesh])’. Algorithms is a broad term including subcategories such as AI, Machine Learning, Natural Language Processing, and Neural Networks, while Sensitivity and Specificity includes subcategories such as Predictive Value of Tests, Roc Curve, And Signal-to-Noise Ratio (online Supplementary Materials 1). Taken together, these terms narrowed the search onto papers which focused on prediction. Fifty-seven references were returned and additional references were hand-searched. Papers which did not report metrics on the accuracy of predictions or did not focus on TRD were excluded, leaving 17 studies for review, with an additional 5 identified through hand search, for a total of 22 studies.

## Results

### Is there a uniform definition of treatment-resistant depression?

The first search yielded a total of fourteen review studies focusing specifically on the topic of how the concept of TRD is defined and operationalized. They either focused on definitions of TRD and staging models (Brown et al., 2019; Demyttenaere & Van Duppen, 2019; Gaynes et al., 2020; Malhi & Byrow, 2016; McIntyre et al., 2014; Ng et al., 2019; Pandarakalam, 2108; Ruhé, Van Rooijen, Spijker, Peeters, & Schene, 2012; Sackeim et al., 2019; Trevino, McClintock, McDonald Fischer, Vora, & Husain, 2014), or on the emerging shift away from the concept of TRD, in favor of the alternative notion of ‘difficult to treat’ depression (Cosgrove, Naudet, Hiogberg, Shaughnessy, & Cristea, 2020; Demyttenaere, 2019; McAllister-Williams et al., 2020; Rush, Aaronson, & Demyttenaere, 2019). All were published after 2012, the year in which PubMed started using the MeSH index term ‘Treatment-Resistant Depression’. Ten of fourteen review studies were published after 2018, indicating that this topic has been subject to recent discussions.

The reviews about definitions of TRD reported on the wide range of current definitions of TRD, and the associated challenges for TRD research and treatment. One systematic review found 155 definitions for TRD among the 150 studies included, with about half (50.3%) requiring at least 2 treatment failures and only a minority (11%) including neuromodulation (Brown et al., 2019). Another review found that only 20% of studies used the most common definition of TRD of at least 2 failed treatments *and* confirmation of prior adequate dose and duration (Gaynes et al., 2020). Importantly, patient-oriented outcome measures focusing on functional impairment or quality-of-life were rarely used.

Reviews about alternative conceptualizations of TRD focused on ways to address the problem of heterogeneity in TRD definitions and concepts. Proponents of the shift to ‘difficult-to-treat’ depression call for a more holistic dimensional focus that includes psychosocial functioning and quality-of-life (McAllister-Williams et al., 2020; Rush et al., 2019). At the same time, others were skeptical about creating a possibly over-inclusive label (Cosgrove et al., 2020). However, proponents and skeptics alike agree that current concepts of TRD have important limitations, notably their biological heterogeneity and their focus on psychopharmacological treatments, with limited data on psychotherapy or neuromodulation.

Hence, although there is no agreed upon definition of TRD, there is agreement that current definitions are limited (primarily designed for psychopharmacological research), and discussions about conceptualization of TRD in research and clinical practice are ongoing.

### Can we predict group-level long-term outcomes of treatment-resistant depression?

We found a total of 5 studies focusing on long-term outcomes of TRD specifically defined as such (Table 1). The focus on TRD and its long-term outcomes in naturalistic settings is relatively recent: a first systematic review was published in 2009 (Fekadu et al., 2009), after which a total of four medium to long-term observational studies were published, all from the same research group (Fekadu et al., 2011, 2012; Vergunst et al., 2013; Wooderson et al., 2014).

**Table 1.** Overview of medium- to longer-term outcome of TRD

Reference	Patient characteristics	Setting	*N*	Outcome	FU period	Predictors	Strengths
1a. First systematic review of 9 studies on medium- to longer-term outcome of TRD.							
Fekadu et al. (2009)	TRD defined as failed response to min.1 antidepressant; *and* one of following: HRS-D25 > 15, MDD in various stages of resistance; HRSD > 18; referred for ECT; residual symptoms or chronic depression	Mostly from secondary/tertiary services; 1 study from outpatient setting	1279	Good outcome (recovery or absence of relapse) in 38%-48% (3 studies) Poor outcome varied between 28% and 68% (3 studies)	Ranging between 1–10 years, but short in most studies (1–2 years)	For good outcome & recovery initial responsiveness to lithium absence history of admission shorter duration of illness & less severe illness during FU For poor outcome & readmission prior history of treatment with lithium presence of delusions & agitation	First systematic review of short- and longer-term outcomes studies of (heterogeneously defined) TRD
2a. Follow-up studies of longer-term outcome in patients with TRD.							
Fekadu et al. (2011)	TRD defined as failed response to min. 1 antidepressant trial; Other: duration of illness ranging between 16–22.2 years	Patients discharged from specialized in-patient treatment unit	118	Measures used: LIFE chart, PSR 69% remission or partial remission <40% full remission at any one follow-up point in time At FU: Remission in 70% of those discharged in remission 50% of those discharged in partial remission 30% of those discharged in episode	Median of 3 years	For long-term outcome: Posttreatment clinical status at discharge (defined by PSR score) AOR 3.1 95% CI 1.91–5.07	First report on longer-term outcome as a function of baseline end of treatment clinical status in explicitly defined TRD patients
Fekadu et al. (2012)	TRD defined using the MSM with mean severity of 10.1 (moderate to severe on average); Other: 65% ECT; mean number of prior AD 5.9; 60% history of suicide attempt (unipolar = 77; bipolar = 27; secondary TRD = 14)	Patients discharged from specialized in-patient treatment unit	118	Main measures used: LIFE chart, PSR At discharge Remission 33.9% Partial remission 30.5% Persistent depressive symptoms 35.6% At FU 48.3% recovery (defined as in remission for min.6 months) 11.9% remission (defined as asymptomatic for min. 1 months) 39.8% persistent depressive symptoms	Ranging from 8 to 84 months; mean 39 months mean HRSD 20.5	For remission during FU Educational achievement HR = 1.17, 95% CI 1.01–1.35; *p* = 0.03 Level of social support HR = 1.76, 95% CI 1.07–2.89; *p* = 0.03 For non-remission during FU Severity of TRD defined by MSM HR = 0.77, 95% CI 0.68–0.99, *p* = 0.04	First report on predictors of longer-term outcome and in explicitly defined TRD patients. Note: Poor social support was independently associated with relapse (HR = 3.55, 95% CI 1.01–12.54; *p* = 0.05). Although not a predictor, the use of MAO-Is while inpatient was independently associated with remission at discharge after controlling for other treatments.
Vergunst (2013)	TRD defined as: failed response to min. 1 antidepressant trial; Other: min. 1 mood stabilizer in 96 (83.5%); prior ECT in 69% (unipolar = 84; bipolar = 31)	TAU after discharge from specialist tertiary unit	115	Main measures used: LIFE chart and PSR At FU: 60.3% asymptomatic or subthreshold level; 39.7% chronic symptoms (15.8% mild; 13.9% moderate; 10%severe)	Ranging from 1–7 years (median 36 months)	For mean symptom severity during FU -Social support (beta −0.356, *p* = 0.001)	Reports analyses predicting symptom severity fluctuations and symptom severity from range of social and clinical variables.
Wooderson et al. (2014)	TRD defined as commensurate with entry criteria for STAR-D (unipolar *n* = 51; bipolar *n* = 20)	Specialist multi- disciplinary treatment	71	Main measure used: HDSR-21 score of 10 or less; CGI At FU Good outcome 66% Intermed/poor outcome 34% Responders 56% Remission 51%	Median 34 months; IQR 19–52	None reported.	First study looking at long-term outcome in TRD subgroups in terms of diagnosis, HDRS21 factors, pattern of response to treatment and psychosis history. Identifies *possible* predictors of response.

The systematic review by Fekadu et al. (Table 1) is the first comprehensive review to incorporate follow-up studies of TRD, including studies which: (1) defined treatment-resistance as a failure to respond to at least one antidepressant or where treatment-resistance could be inferred from the overall description, (2) were longitudinal (3) had a minimum duration of 6 months (i.e. going beyond the usual short-term follow-up as part of an acute treatment trial) (4) used defined dimensional or categorical outcomes. The authors reviewed 9 studies (including a subsample of the well-known STAR-D study) for a total of 1279 participants. In all but one study, patients were recruited from secondary and tertiary services, but most patients had a chronic history of severe illness. Of the two largest studies, patients included had either chronic major depression of at least 4 previous episodes (Dunner et al., 2006), or a history of recurrent depression in 74.7%, with mean duration of illness of 15.3 years and mean age at first episode of 25.5 (Rush et al., 2006).

The largest study showed a cumulative remission rate of 70% at one-year follow-up (Rush et al., 2006). Other studies found a ‘good outcome’ (i.e. recovery or the absence of relapse) in 38–48% (3 studies) and a ‘poor outcome’ (i.e. relapse or premature death) varying between 28–68% (3 studies). Overall, the review found that TRD is a highly relapsing condition, with substantial disability and mortality. However, duration of follow-up was short in most studies. In fact, the two largest studies had a follow-up period of 1 and 2 years, respectively, and both studies used a very short duration to define relapse (1 week) (Dunner et al., 2006; Rush et al., 2006). The review leaves open the possibility that, based on longitudinal studies of affective disorders, outcomes might have been better if longer duration of follow-up had been used, as seen in a 12-year follow-up safety study (Nugent, Iadarola, Miller, Luckenbaugh, & Zarate, 2016). Finally, only two studies reported on social outcomes like quality-of-life or functioning.

Since the above systematic review, four studies have been published (2011–2014); these were the first follow-up studies to recruit participants *explicitly defined* as having TRD (Table 1). Although treatment-resistance was defined as a failed response to at least 1 antidepressant, the patients' severity of illness at entry was significant with a moderately severe to severe TRD (per the Maudsley Staging Method) (Fekadu et al., 2012), a mean duration of illness of 16–22.2 years (Fekadu et al., 2011), and treatment history of ECT in 69% (Vergunst et al., 2013) or prolonged intensive multidisciplinary inpatient therapy with a minimum score of 16 on the 21-item Hamilton Depression Rating Scale (Wooderson et al., 2014). Overall, sample sizes were relatively small, ranging from 71 to 118, and two of the four studies involved the same set of participants (Fekadu et al., 2011, 2012).

These four studies reported on longer-term outcomes (mean of 3 years) in patients with TRD. The first study found that 69% achieved remission or partial remission, with outcomes at follow-up (median of 3 years) varying according to the status at discharge (Fekadu et al., 2011). The second study found that at follow-up (mean of 39 months), 60.2% reached full remission, with 39.8% showing persistent depressive symptoms (Fekadu et al., 2012). This study reported on *predictors* of longer-term outcome in TRD patients. Higher educational achievement (hazard ratio (HR) = 1.17, 95% CI 1.01–1.35; *p* = 0.03) and strong level of social support (HR = 1.76, 95% CI 1.07–2.89; *p* = 0.03) were found to be predictors of remission during follow-up. The third study showed similar outcomes at follow-up: 60.3% were asymptomatic or at subthreshold level and 39.7% had chronic symptoms (Vergunst et al., 2013). Of the tested predictors of mean symptom severity (e.g. social support, number of prior of depressive episodes, duration of admission), only social support was found to be a significant predictor (beta −0.356, *p* = 0.001). The fourth study found that, with intensive multidisciplinary treatment, 66% had a good outcome and 18–34% had poor to intermediate outcome at follow-up (median of 34 months) (Wooderson et al., 2014). The study showed that patients can maintain clinical improvement 3 years (mean) post-discharge following intensive multidisciplinary TRD treatment.

These four longitudinal studies build on emerging evidence about long-term outcomes of TRD (Fekadu et al., 2009). Despite the significant severity of depression and chronicity of treatment-resistance upon study entry (mean duration of 16 to 22 years), a majority achieved remission, while a substantial minority had persistent depressive symptoms. This raises a separate question, namely whether physicians can reliably distinguish those who will recover from those who will not, on an *individual*, rather than *group-level,* basis.

### Can we make individual predictions of treatment-resistance in depression?

We found 22 studies investigating individual prediction of treatment-resistance in depression. Thirteen studies (Table 2.A) focused on whether an individual patient who has failed to respond to multiple past treatments will respond to the next treatment. These studies were relatively recent, with small sample sizes. The remaining nine studies (Table 2.B) focused on the question of which patients with major depression will develop treatment-resistance (defined in a variety of ways).

**Table 2.** Individual prediction of treatment-resistance in depression

Citation	Purpose	Sample (Trial)	Disorder at inclusion	Intervention	Trying to predict…	Definition of TRD (non-response or resistance)	Prediction endpoint	Predictors/features
Section A. Predicting TRD patients' response to additional treatment								
Bailey et al. (2018)	Among treatment resistant patients, use baseline EEG and clinical measures to predict who will respond to TMS.	39 TRD patients	TRD as defined by Stage 2 of Thase and Rush classification; HAM-D > 20	5–8 weeks of rTMS	Responders (metrics recalculated)	<50% reduction in HAM-D	At the end of treatment	16 EEG, 3 mood, and 6 behavioral features
Bailey et al. (2019)	Among treatment resistant patients, use baseline EEG and clinical measures to predict who will respond to TMS.	42 TRD patients	TRD as defined by Stage 2 of Thase and Rush classification; HAM-D > 20	5–8 weeks of rTMS	Responders (metrics recalculated)	<50% reduction in HAM-D	At the end of treatment	53 EEG variables and 1 clinical (MADRS)
Bares et al. (2017)	Among treatment resistant patients, use one EEG and one clinical measure to predict response to SSRI treatment.	38 TRD patients	‘at least’ Stage I according to Thase and Rush; MADRS ≥ 25 and CGI ≥ 4.	6-weeks of SSRI treatment	Responders (metrics recalculated)	At least 50% reduction in MADRS score	At the end of treatment	Combination of reduction in MADRS ≥ 20% at week 2 and EEG decrease of cordance at week 1 compared to baseline
Bares et al. (2015)	Among treatment resistant patients, use one EEG and two clinical measures to predict response to SSRI treatment.	87 TRD patients	‘at least’ Stage I according to Thase and Rush; MADRS ≥ 25 and CGI ≥ 4.	At least 4-weeks of antidepressant treatment	Responders (metrics recalculated)	At least 50% reduction in MADRS score	At the end of treatment	Combination of reduction in MADRS ≥ 20% at week 1, reduction in MADRS ≥ 20% at week 2, and EEG reduction of cordance value at week 1 compared to baseline
Carrillo et al. (2018)	Among treatment resistant patients, use average negative and positive words during initial interview to predict who will respond to psilocybin.	17 TRD patients	Resistance as defined by ≥ 17 on HAM-D and failure at least 2 AD trials	2 doses of psilocybin	Responders (metrics recalculated)	<50% reduction in QIDS	5 weeks post-treatment	2 features: average positivity and average negativity during baseline autobiographical memory test
Ge et al. (2017)	Among treatment resistant patients, use fMRI data from one brain region to predict response to rTMS.	18 TRD patients	Failure to achieve clinical response to an adequate dose of an antidepressant based on an Antidepressant Treatment History Form (ATHF) score of ≥ 3 OR unable to tolerate at least 2 separate trails of antidepressants of inadequate dose and duration (ATHF 1 or 2); AND HDRS = 17 ≥ 18	4–6 weeks of rTMS	Responders (metrics recalculated)	At least 50% improvement in HRSD	At the end of treatment	dACC in the SN from fMRI
Kautzky et al. (2015)	Among depressed patients, use combination of SNPs and clinical variables to predict treatment resistance after 1 antidepressant trial.	225 MDD patients (from GSRD)	MDD, diagnosed according to DSM-IV criteria	At least 1 antidepressant trial (most received more than one)	Responders (metrics recalculated)	HAM-D > 17 after at least one AD trial	At the end of treatment	3 SNPs and 1 clinical variable (melacholia)
Khodayari-Rostamabad et al. (2013)	Among treatment resistant patients, use baseline EEG measures to predict who will respond to an additional antidepressant trial.	22 TRD patients	TRD as defined by failure to respond to at least 2 previous antidepressant trials and HAM-D ≥ 18	6 week SSRI treatment	Responders (metrics recalculated)	<30% improvement between pre/post HAM-D	At the end of treatment	Pre-treatment EGG measures
Micoulaud-Franchi et al. (2012)	Among treatment resistant depressed and bipolar patients, use one EEG measure to predict response to rTMS.	21 treatment resistant MDD and BD patients	Non-response to pharmacological treatment of depression using a minimum of 2 distinctly different classes of antidepressant medications	20 rTMS sessions over 4 weeks	Responders (metrics recalculated)	At least 50% reduction of baseline BDI scores	At the end of treatment	EEG pre-treatment alpha band power in the right
Minelli et al. (2016)	Among treatment resistant patients, use seizure quality to predict response to ECT.	45 TRD patients	Failure to respond to at least 2 antidepressant trials of different classes AND to an adequate trial of a tricylic (TCA) (Stage III of Thase and Rush)	ECT therapy 3 times per week until considered complete by judgment of treating physicians	Responders (metrics recalculated)	At least 50% reduction in MADRS score	1 month after the end of treatment	Seizure quality of the 4th and 6th ECT sessions, as rated by 2 doubled blinded independent psychiatrists
Richieri et al. (2011)	Among treatment resistant patients, use composite score from brain SPECT to predict response to rTMS.	33 TRD patients	non-response to pharmacological treatment of depression using a minimum of 2 distinctly different classes of antidepressant medications	20 rTMS sessions over 4 weeks	Responders (metrics recalculated)	At least 50% reduction in baseline BDI scores	At the end of treatment	Composite score for whole-brain voxel-based regional cerebral blood flow (rCBF) from baseline brain SPECT
Sun et al. (2016)	Among treatment resistant patients, predict remission of suicidal ideation using EEG measures in the prefrontal cortex to predict response to MST.	27 TRD patients	‘Quantified with the antidepressant treatment history form’. they do not specify in the methods if a cut-off score was used. in the introduction, they define TRD as inability to respond to 2 or more separate trials of antidepressants	24 sessions of magnetic seizure therapy or until remission of depressive symptoms* *(defined as HRSD ≤ 10 and 60% reduction in symptoms for at least 2 days)	Remission of suicidal ideation (metrics recalculated)	SSI score of 0	At the end of treatment	Baseline TMS-EEG measures 1 week before MST – measures of cortical inhibition (e.g. N100 and LICI) in the frontal cortex
van Waarde et al. (2015)	Among severely depressed and/or treatment resistant patients, use baseline fMRI measures to predict response to ECT treatment.	45 severe MDD/TRD patients	‘Severe and/or treatment-resistant depression as diagnosed by at least 2 independent experienced psychiatrists’ according to the DSM-IV; does not explain how treatment resistance is defined	2 weekly ECT sessions for up to 10 weeks	Responders (metrics recalculated)	MADRS > 10	At the end of treatment	Resting-state networks from MRI and fMRI
Section B. Predicting depressed patients' development of TRD								
Dinga et al. (2018)	Among MDD patients, use mood, behavioral, and EEG measures to predict who enters rapid remission v. gradual improvement v. chronic depression (TRD).	804 MDD or dysthymia patients (from NESDA)	MDD or dysthymia patients	Any pharmacological or psychotherapeutic treatment or no treatment	TRD	3 classification groups formed by latent class growth analysis, where TRD is the ‘chronic’ group (and the other two groups are: rapid remission; gradual improvement)	2 years after treatment	81 clinical variables, personality dimensions, demographic variables, and biological variables (BMI, inflammatory markers, metabolic syndrome variables, vitamin D levels, and more)
Chang et al. (2019)	Among depressed patients, predict which of 14 antidepressants (or 91 combinations of antidepressants) will decrease a patient's depression scores the most.	121 MDD patients 13 MDD patients for external validation	MDD, not specified	At least 1 antidepressant trial	Responders (metrics recalculated)	<50% reduction in HAM-D	At the end of treatment	127 demographic features, 20 neuroimaging biomarkers, 20 genetic variants, and 20 DNA metylation features chosen from elastic net feature selection. (Antidepressant info is accounted for in one of the other neural network layers)
Cepeda et al. (2018)	Using medical claims records of depressed patients, use clinical features of drug utilization to predict which patients will later receive ECT, DBS, or VNS (as a proxy for TRD).	22 057 patients in the CCAE insurance claims database 14 845 patients in two other insurance claims databases for external validation	MDD or other depression diagnosis	At least 1 antidepressant in the past year	TRD (proxy)	Patients with a procedure code on inpatient or outpatient medical claims record for electroconvulsive therapy (ECT), deep brain stimulation (DBS), or vagus nerve stimulation (VNS)	Up to 1 year after initial AD prescription	10 features involving drug utilization and number of therapy sessions extracted from claims
Perlis et al. (2012)	Using medical records and billing codes of depressed patients, predict whether a patient was depressed or well during each visit, and then classify whether they are treatment-resistant based on the ratio of well to depressed visits during antidepressant trials over a 1-year period.	5198 MDD patients from out-patient psychiatry medical records and billing codes	At least one billing code with diagnosis as MDD	At least 1 antidepressant trial within a 12-month period	TRD	Machine learning was used to classify each visit as either depressed or well or intermediate. those classified with ‘TRD’ had to meet the following criteria: 2 + depressed visits within 12 months following an initial AD prescription, no well visits, a majority of all visits classified as depressed, and exposure to at least 2 ADs during this period	Up to one year after first AD prescription	34 features from natural language processing of medical records and billing codes
Kautzky et al. (2017)	Among depressed patients, use combination of clinical, sociodemographic, and psychosocial variables to predict treatment resistance after 2 antidepressant trials	400 MDD patients (from GSRD) 80 MDD patients for external validation	MDD, diagnosed according to DSM-IV criteria	2 antidepressant trials	TRD	HDRS ≥ 17 after at least 2 AD trials	At the end of treatment	48 clinical features
Kautzky et al. (2018)	Among depressed patients, use 47 clinical and sociodemographic features to predict whether patients will respond to their second antidepressant treatment for the current depressive episode.	552 MDD patients (from GRSD) 119 MDD patients for external validation	MDD, diagnosed according to DSM-IV criteria	2 antidepressant trials	TRD	<50% reduction in MADRS and MADRS ≥ 22	At the end of treatment	15 clinical features (the top 15 predictors taken from the initial 47)
Kautzky et al. (2019)	Among depressed patients, use 16 clinical features to predict who will respond to their second antidepressant treatment for the current depressive epsiode.	602 MDD patients (from GRSD's TRD-III) 314 MDD patients for external validation (from GRSD's TRD-I)	MDD, diagnosed according to DSM-IV criteria	2 antidepressant trials	TRD	TRD-III: < 50% reduction in MADRS and MADRS ≥ 22 TRD-I: HAM-D ≥ 16	At the end of treatment	16 clinical features
Perlis (2013)	Among depressed patients, use clinical variables to predict who will respond after 1–2 antidepressant trials *v.* who will not respond after 2.	2094 MDD patients (from STAR*D) 461 MDD patients for external validation	MDD, diagnosed according to DSM-IV criteria	Sequential treatment levels beginning with citalopram for 12 weeks, then moving to randomized next level if still not remitted.	TRD	QIDS-SR > 5	A the end of treatment	15 clinical variables chosen from initial 48 during feature selection
Nie et al. (2018)	Among depressed patients, use nearly 700 diverse features to predict who will not respond after 2 antidepressant treatment trials.	2454 MDD patients (from STAR*D) 225 MDD patients for external validation (from RIS-INT-93)	STAR-D: met DSM-IV criteria for MDD RIS-INT-93: met DSM-IV criteria for MDD and ‘had history of resistance to therapy with AD medication’	STAR*D: went through 4 levels of treatment options, for up to 12 weeks each RIS-INT-93 cohort: treated with citalopram for up to 6 weeks	TRD	STAR*D: > 5 on QIDS-C or QIDS-SR RIS-INT-93: > 7 on HAM-D	At end of treatment	Began with 700 clinical features; for validation, used set of 22 overlapping features

Citation	Methodology	Form of validation	Flipped? (*)	AUC	Sens.	Spec.	PPV	NPV	Acc.	Bal. Acc	F1	Other
Section A. Predicting TRD patients' response to additional treatment												
Bailey et al. (2018)	Linear support vector machine (SVM) classifier with 5-fold cross-validation	Cross-validation	Yes		0.92*	0.91*				0.91	0.93	10 responders of 39, prevalence = 0.2564
Bailey et al. (2019)	Linear support vector machine (SVM) classifier with 5-fold cross-validation	Cross-validation	Yes		0.89*	0.84*				0.866		12 responders of 42, prevalence = 0.2857
Bares et al. (2017)	Inferential statistics with ROC analysis	None	Yes	0.92			0.71*	0.95*	0.84			Number needed to diagnosis = 1.4
Bares et al. (2015)	Inferential statistics with ROC analysis	None	Yes	0.91			0.8*	0.85*	0.83			Number needed to diagnosis = 1.53
Carrillo et al. (2018)	Gaussian Naive Bayes classifier with 7-fold cross-validation	Cross-validation	Yes					0.75*	0.85			
Ge et al. (2017)	Inferential statistics with ROC analysis	None	Yes	0.939	0.82*	1*			0.939			11 responders of 18, prevalence = 0.6111
Kautzky et al. (2015)	Random forest classifier with 10-fold cross-validation	Cross-validation	Yes			0.25*		0.5*				
Khodayari-Rostamabad et al. (2013)	Mixture of Factor Analysis (MFA) classifier with leave-n-subjects-out (LnO) cross-validation	Cross-validation	Yes		0.8093*	0.9486*			0.879			7 responders of 22, prevalence = 0.3182
Micoulaud-Franchi et al. (2012)	Inferential statistics with ROC analysis	None	Yes	0.815	0.66*	1*	1*	0.8*				Cut point = 1.49 mu-V
Minelli et al. (2016)	Inferential statistics with ROC analysis	None	Yes	0.74	0.688*	0.897*	0.615*	0.828*				Cut point = medium quality seizure
Richieri et al. (2011)	Inferential statistics with ROC analysis	None	Yes	0.89	0.73*	0.94*	0.92*	0.81*				
Sun et al. (2016)	Inferential statistics with ROC analysis	None	Yes	0.9	0.89*	0.9*			0.89			Authors reported remission rate as 53%.
van Waarde et al. (2015)	Support vector machine (SVM) classifiers with leave-one-per-group-out cross-validation	Cross-validation	Yes		0.85*	0.84*		0.88*				
Section B. Predicting depressed patients' development of TRD												
Dinga et al. (2018)	Multinomial generalization of penalized elastic net logistic regression classifiers with 10-fold cross-validation	Cross-validation	No	0.66	0.47					0.61		
Chang et al. (2019)	Neural network architecture with a patient layer, AD prescription layer, and prediction layer.	External validation	Yes		0.875*	0.8*		0.8*	0.846		0.8	
Cepeda et al. (2018)	Decision-tree classifiers with 80–20 train-test split, no cross-validation	External validation	No	0.79								
Perlis et al. (2012)	Logistic regression classifier with adaptive LASSO procedure and 3-fold cross-validation	Cross-validation	No						0.764			
Kautzky et al. (2017)	Random forest classifier with 10-fold cross-validation	External validation	No		0.633	0.8	0.665	0.784	0.737			
Kautzky et al. (2018)	Random forest classifier with 10-fold cross-validation	External validation	No		0.803	0.603	0.819	0.603	0.706			0.396
Kautzky et al. (2019)	Elastic net logistic regression classifier with 10-fold cross-validation	External validation	No		0.857	0.875	0.793	0.917	0.869			0.124
Perlis (2013)	Of 4ML approaches using 10-fold cross-validation, logistic regression classifier performed the best	External validation	No	0.719	0.259	0.911						
Nie et al. (2018)	Of 5 ML approaches using 10-fold cross-validation, random forest classifier performed the best	External validation	No	0.86	0.92	0.36	0.92	0.39	0.73			

*Note: Sensitivity and specificity and PPV and NPV were recalculated for some studies so that all metrics reflect prediction of TRD as the positive class.

Of the 13 studies focusing on patients with demonstrated treatment-resistance (Table 2.A), all but two had under fifty participants and several involved machine learning (Bailey et al., 2018, 2019; Bares, Novak, Brunovsky, Kopecek, & Höschl, 2017; Bares et al., 2015; Carrillo et al., 2018; Ge et al., 2017; Kautzky et al., 2015; Khodayari-Rostamabad, Reilly, Hasey, de Bruin, & Maccrimmon, 2013; Micoulaud-Franchi et al., 2012; Minelli et al., 2016; Richieri et al., 2011; Sun et al., 2016; van Waarde et al., 2015). Most investigated whether patients would respond to one specific intervention (e.g. TMS, psilocybin). When predictive values were reported, predictions that a patient would not respond to the specific intervention tested varied, with accurate predictions ranging from 61.5% (total *N* = 45) to 100% (total *N* = 21) (Micoulaud-Franchi et al., 2012; Minelli et al., 2016). The nine studies focusing on which patients with major depression might develop TRD (broadly defined) are more extensive and include large multi-site trials of hundreds or thousands of patients, with a wide variety of predictors (Table 2.B). These studies vary by design and study size, and include: (1) pragmatic trials, i.e., reflecting real-world conditions, (2) large sampled, regimented trials involving large datasets like the STAR*D dataset, and (3) studies using medical records.

Firstly, the two pragmatic trials involved available treatment for depression (Chang et al., 2019; Dinga et al., 2018). One study followed 804 MDD or dysthymia patients receiving any combination of pharmacological, psychotherapeutic, or no treatments (Dinga et al., 2018). This study, based on the Netherlands Study of Depression and Anxiety dataset, covered a wide range of illness severity. The model predicted who would develop TRD (defined as chronic depression with no improvement after two years of any or no treatments), found that about half (47%) of the TRD patients were correctly predicted to be so. This is the only prediction model built on naturalistic study data that we found, and the study with the longest prediction endpoint. However, it lacks external validation. The second study involved a network approach to antidepressant resistance with 121 patients (Chang et al., 2019). In a small testing dataset (*N* = 13) of patients with MDD, 80% of the patients who were predicted to respond to treatment did in fact respond to their prescribed antidepressants. A network was designed to output modeling about the predicted effectiveness of antidepressants for every patient, and outperformed baseline models both for prediction of response and of remission.

A second set of five studies, involving large STAR*D or GRSD (Group for the Study of Resistant Depression) datasets, identified which depressed patients would not respond to their second (Kautzky et al., 2017, 2018, 2019; Perlis, 2013) or subsequent (Nie, Vairavan, Narayan, Ye, & Li, 2018) antidepressant trial. Samples ranged from 400 to 2454 patients, with large external validation samples. The models' predictive accuracies during validation were variable: for predictions that a patient would respond to a subsequent antidepressant (i.e. symptom reduction), the models were correct from 39% (*N* = 225) to 81.9% (*N* = 314) of the time. For predictions that a patient would not respond to a subsequent antidepressant, the models' accuracy ranged from 66.5% (*N* = 80) to 92% (*N* = 225).

A third set of two studies used patient records to predict treatment-resistance (Cepeda et al., 2018; Perlis et al., 2012). One study used insurance claims of 22 057 patients to predict which patients would receive neuromodulation after trying an antidepressant in the past year (Cepeda et al., 2018). The authors found that their algorithmically-derived decision-tree rule performed more accurately in internal validation than any of the five decision rules defined by expert psychiatrists (*F*-1 = 0.44 compared to *F*-1′s = 0.39–0.42) and held up in external validation samples totaling 14 845 patients from alternate insurance databases (*AUC*s = 0.78–0.79). A second study used natural language processing of 5198 patient records to develop a model predicting whether patients were depressed on every given visit following an antidepressant prescription (Perlis et al., 2012). The authors classified individual visits as depressed (*v.* well) with a positive predictive value of 78%. Next, patients were classified as treatment-resistant if they had a majority of predicted-depressed visits despite 2 antidepressant trials in the past year. Agreement between the model's predictions of treatment-resistance and the opinion of a board of expert clinicians was 76.4%. However, these studies used somewhat unusual endpoints for treatment resistance.

In sum, there is a growing body of evidence assessing the accuracy of predictions about treatment-resistance. Most of the studies are limited by unconventional definitions of treatment-resistance or the use of limited interventions. Studies predicting whether a patient who failed to respond to multiple past treatments will respond to the next treatment are relatively new, underpowered, and lack external validation. Studies investigating whether a patient will *develop* treatment-resistance are more developed, with larger sample sizes, more comprehensive sets of predictors, and larger external validation datasets. Predictive accuracy across a range of metrics varies widely, with the largest and best validated studies showing lower predictive abilities.

## Discussion

Irremediability is a key eligibility requirement for psychiatric EAS and is defined as the lack of reasonable alternatives, which must be ‘assessed in light of the diagnosis and prognosis’ (Euthanasia Code, 2018). While current frameworks allow for a person to refuse a treatment option, guidelines emphasize a default objective standard for irremediability (NVVP, 2018; VVP et al., 2017). Whether clinicians *can* accurately determine, as per ‘current medical understanding’, prognosis and irremediability in the context of psychiatric EAS is a key question in the debate about irremediability – and the central question we examined here. Given that debates about irremediability hinge on the key issue of ‘objective *v.* subjective’ standard of irremediability, whether the objective standard of irremediability in psychiatry is relevant is of crucial importance for policymaking around the world, for ongoing and future discussions about extending EAS laws to include psychiatric EAS.

### Discussion of main findings

Although the term TRD has gained wide use, it is used primarily for research purposes and relatively recently: over 150 definitions exist, and active discussions about appropriate outcome measures for TRD are ongoing. Unlike what it seems to suggest, ‘treatment-resistance’ does not mean that there are no remaining options, and definitions evolve with the introduction of new treatments (e.g. esketamine, NNT of 5) (Kasper, 2022). At the same time, scientific knowledge about group-level long-term outcomes of TRD is limited. Four naturalistic studies focused on medium to long-term outcomes in patients who were explicitly defined as having TRD at beginning of follow-up (Fekadu et al., 2011, 2012; Vergunst et al., 2013; Wooderson et al., 2014). These studies showed that included patients, despite being well-characterized as treatment-resistant at the onset of the studies and after having received years of community treatment – i.e., persons with extensive psychiatric histories, comparable those requesting and receiving psychiatric EAS currently (Kim, De Vries, & Peteet, 2016; Nicolini, Peteet, Donovan, & Kim, 2020b; Thienpont et al., 2015) – a majority significantly improved – i.e., reached remission. Furthermore, they found a role for non-biological predictors such as education level or social support in TRD outcomes. The limitations of these studies included: (a) their small number overall, (b) their small sample sizes with internal overlap in terms of participants, (c) their focus on TRD defined primarily as failed pharmacological treatments, (d) the absence of newer agents with proven efficacy for TRD, and (e) their overall limited usefulness for *individual* outcome prediction.

Individual prediction studies were found to have overall modest predictive ability, were often not tested in prospective studies, and limited applicability in practice. Studies focusing specifically on response prediction in patients with TRD were relatively limited in size and scope, involving only specific treatments (e.g. TMS, psilocybin), and focusing on experimentally relevant predictors (e.g. ECT seizure quality). Among the larger and more rigorous studies of patients with major depression, the models' predictive ability is unlikely to be sufficient for clinical use. Overall, the individual prediction studies had the following limitations: (a) most models only predicted whether patients will respond to a particular treatment rather than all available treatments (and if so, to which of available treatments), (b) only *one* study involved long-term follow-up of sustained remission (Dinga et al., 2018), (c) potential wrongful inflation of accuracy estimates (e.g. related to small sample sizes, absence of testing model performance in an external sample, and problematic validation methods), precluding reliable immediate implementation in clinical practice (Hosseini et al., 2020; Jacobucci, Littlefield, Millner, Kleiman, & Steinley, 2020; Poldrack, Huckins, & Varoquaux, 2020). Finally, the model which came closest to reflecting real-life conditions (Dinga et al., 2018), accurately predicted outcomes (i.e. who would continue to have chronic depression after two years of any or no treatments) in only 47% of cases – that is, at chance level.

### Implications for the debate about irremediability in psychiatric EAS

The findings of this scoping review raise several implications for the debate about irremediability in psychiatric EAS. First, our findings show that the objective standard for irremediability will be difficult to meet, at least in the paradigm case of depression, because a clinician cannot accurately determine irremediability, as argued by many (Appelbaum, 2017; Blikshavn et al., 2017; Broome & de Cates, 2015; Cowley, 2013, 2015; Jansen, Wall, & Miller, 2019; Kelly, 2017; Kelly & McLoughlin, 2002; Kim & Lemmens, 2016; Kissane & Kelly, 2000; Miller, 2015; Naudts et al., 2006; Olié & Courtet, 2016; Schoevers, Asmus, & Van Tilburg, 1998; Simpson, 2018; Steinbock, 2017; Vandenberghe, 2011, 2018). Our findings point to the fact that in psychiatric disorders, unlike in somatic disorders, lack of treatment-response does not necessarily entail lack of long-term recovery. This further shows that, in professional debates about irremediability, invoking the construct of TRD is *not* ‘a good starting point for identifying an irremediable psychiatric condition’ (Rooney et al., 2018). Given that a diagnosis of TRD is clearly not sufficient to establish irremediability, the concepts of ‘treatment-resistance’ and ‘irremediability’ should not be conflated.

Second, our findings do not support the claim, made by some, that clinicians can rely on existing statistical and staging tools like the Maudsley Staging Method to predict chances of recovery in a person requesting psychiatric EAS (Provencher-Renaud, Larivée, & Sénéchal, 2019; Rooney et al., 2018; Tanner, 2018). Unlike in somatic medicine, staging methods used for depression do not correlate with prognosis. The fact that a majority of patients with severe chronic depressive illness and high scores on the Maudsley Staging Method – i.e., patients with history similar to those who currently request and receive psychiatric EAS – will enter remission, shows that high disease severity or chronicity does not correlate with long-term symptom persistence or a lack of recovery. Furthermore, potentially promising statistical tools, like machine learning models for individual prediction, although promising, cannot yet be reliably implemented in clinical practice. The best proxy model shows a prediction accuracy at chance level, suggesting that, as things stand, precision psychiatry cannot yet resolve the problem of prognosis prediction in psychiatry.

Finally, our findings provide preliminary evidence for the claim that non-biological social factors, e.g. social support, can affect chances of recovery in psychiatry (Blikshavn et al., 2017; Cowley, 2013; Jansen et al., 2019; Kelly, 2017; Kissane & Kelly, 2000; Miller & Appelbaum, 2018; Pearce, 2017; Schoevers et al., 1998). The role of social support is especially relevant for psychiatric EAS, as loneliness and social isolation are reported in over *half* of Dutch psychiatric EAS cases (Kim et al., 2016), and described explicitly as one of the reasons for requesting psychiatric EAS in a Belgian qualitative study (Verhofstadt, Thienpont, & Peters, 2017). The role of social factors points to the key issue of explanatory pluralism in psychiatry (Gardner & Kleinman, 2019; Kendler, 2019) – a foundational question of clear ethical relevance for the debate about psychiatric EAS.

### Future research

The debate about irremediability in psychiatric EAS needs clarity about whether it adheres to an objective or a subjective standard for irremediability. Our findings show that for the paradigm case of TRD, as things stand, the objective standard for irremediability in psychiatric EAS fails, and points to several avenues for future research.

On the objective standard for irremediability, there is an open *empirical* question of how reliable prediction psychiatry will be regarding long-term outcomes and responses to (a list of) available evidence-based treatments. In addition, there is an open *policy* question of what an acceptable threshold for reliability might be. Our findings point to avenues that inform the former. First, we need more large-sampled naturalistic and prediction psychiatry studies looking at long-term outcomes, both at the group-level and individual-level. Second, given that persons requesting psychiatric EAS often have psychiatric comorbidities, notably personality disorders (Kim et al., 2016; Nicolini et al., 2020b; Thienpont et al., 2015), trials that include the effect of comorbidity on long-term outcomes are crucial. Third, predictors of outcomes need to include a range of clinical (biological and psychological) *and* social predictors, in a way that aligns with the recognized explanatory pluralism in psychiatry.

While this empirical research might further our conception of the objective standard for irremediability, which standard should prevail – objective or subjective– is a separate question, one that cannot be settled by empirical evidence. Further normative debate is needed to determine whether a subjective standard should prevail and if so, how it should be conceived of – issues beyond the purview of this paper.

### Strengths and limitations

This paper is the first to comprehensively examine the scientific evidence about what we mean by ‘treatment-resistant’ – using depression as a test case – and the implications for debates about irremediability in psychiatric EAS. The fact that long-term follow-up studies included TRD patients with chronic and severe illness makes it especially relevant for the context of psychiatric EAS. The paper has several limitations. First, we chose TRD as a focus as this has been the paradigm case within the debate on irremediability. The results remain thus limited to TRD. However, this type of review can be applied to other psychiatric disorders such as schizophrenia or bipolar, e.g. using available evidence for prediction algorithms (Alonso et al., 2018). Second, our scoping review involved only one database. Finally, our findings clarify what we mean by irremediability when this includes a medical judgment, as emphasized by prevailing guidelines for psychiatric EAS evaluations. For those who emphasize a subjective interpretation of irremediability – i.e., that it is what the patient defines as irremediable – our findings provide a rigorous evidence-based picture of the objective standard for irremediability, that can be juxtaposed against the subjective standard.

## Conclusion

Irremediability remains at the center of debates about the practice of EAS for psychiatric disorders, with main disagreement about whether clinicians can reliably assess irremediability in psychiatry. Using TRD as a test case, we find that current evidence does *not* support the view that clinicians can accurately predict long-term chances of recovery in a particular person with TRD, nor that statistical and staging tools can be used for reliable assessments of irremediability. Our findings suggest that the objective standard for irremediability in psychiatric EAS cannot be met, raising implications for policy and practice around the world.

## Supplementary material

For supplementary material accompanying this paper visit https://doi.org/10.1017/S0033291722002951. click here to view supplementary material
