# Supplementary material for: Irremediability in psychiatric euthanasia: examining the objective standard
Source: Psychol Med. 2022 Oct 28;53(12):5729–47. doi: 10.1017/S0033291722002951 (PMC10482705; doi:10.1017/S0033291722002951)
Supplement: Supplementary file 1 [file S0033291722002951sup.zip › S0033291722002951sup001.docx]

**Supplementary Materials 1. Glossary Individual Prediction**

**Section A: Some frequently used terms and their explanation**

| Artificial Intelligence | Broadly defined, the use of advanced computer programming and mathematics to design automated forms of human intelligence. |
| --- | --- |
| Algorithm | Used to refer to the product(s) of any mathematical model produced by a form of artificial intelligence. For example, ‘algorithm’ can be synonymous with ‘machine learning model’. |
| Machine Learning | An umbrella term for many types of artificial intelligence wherein computers identify complex patterns in data and use these patterns to ‘learn’ how to steadily improve a task without human intervention. |
| Supervised Machine Learning | A form of machine learning where the pattern identified in the model (e.g., the ‘learning’) is confirmed or denied by corresponding data, allowing for estimates of model’s performance or accuracy. See Section B below. |
| Natural Language Processing | A form of artificial intelligence wherein computers are trained to identify and understand human language. |
| Neural Networks | A form of advanced machine learning, also called ‘deep learning’, built to mimic the structure and complexity of the human brain. Neural networks involve several layers of modeling and are therefore considered more complex than most forms of supervised machine learning. |
| Sensitivity | See Section C below |
| Specificity | See Section C below |
| Predictive Value | See Section C below |
| ROC Curve | A plot of the true positive rate against the false positive rate of a classification (e.g., disease vs no disease). Generally used to compare the accuracy of a diagnostic tool or test. |
| Signal-to-Noise Ratio | There is always a chance that datasets will include some patterns or correlations which occur by random chance alone (e.g., do not represent a real pattern or correlation). These random errors are considered “noise” in the dataset.  Additionally, we typically hope that datasets will include some patterns or correlations that reflect real-world differences. These correlations are considered to be a ‘signal’ of the effect in the dataset. Most statistical analyses, including machine learning, aim to determine to what extent patterns or correlations in the dataset reflect true real-world patterns (‘signal’) versus random error (‘noise’). A higher signal-to-noise ratio denotes a more significant effect. |

Adapted from “What Is artificial intelligence?” *Built In* (2019). Retrieved from <https://builtin.com/artificial-intelligence>.

**Section B: How Supervised Machine Learning Works**

| Study Population | Ideally, collect data from hundreds of patients. |
| --- | --- |
| Feature Selection | Decide what predictors (or ‘features’) should be entered into the prediction model. Typically begins with variables previously identified in clinical literature as having predictive value, but also includes other predictors which have not been well-investigated.  Some authors select a set of features and build a model using all of them. Others start with a large set of features (sometimes 100’s), and use a data-driven approach for feature selection, which is an iterative process where the best (most predictive) features are chosen while other (non-predictive) features are dropped from the model. |
| Training the Model | A subset of data is used to ‘train’ the algorithm. Data are labelled -- the dataset includes whether or not each patient responded to the treatment (‘outcomes’). There are many mathematical techniques for this ‘training’ (e.g., support vector machine, k-nearest neighbors, random forest), but most utilize some form of error calculation to identify patterns in the data that predict outcomes. |
| Testing the Model | A subset of the data is used to ‘test’ the algorithm. Data are unlabeled -- e.g., the dataset includes all the predictors for each patient, but does not include whether or not the patient responded to the treatment. The algorithm makes predictions as to whether or not the patient will respond to treatment based off of what it learned in the training. Then, the model’s predictions of outcomes are compared to the true outcomes of each patient. Measures of model accuracy comparing predictions to actual outcomes can then be computed. |
| Cross-Validation | An iterative process where the subsets of data used for training and testing are changed over different subsections (‘folds’) of data until every subset of data has been used for testing at least once. Cross-validation provides some insurance against overfitting. |
| External validation | After the model is trained and tested, there may still be some concern about overfitting. Many researchers recommend that, even after cross-validation, it is important to validate the model’s performance on a set of data it has never been trained on before.  Sometimes, this external validation is a set of data that was collected in tandem with the other data, but was ‘held-out’ of training (and of cross-validation). Other times, the validation set is a completely different dataset collected on a different protocol or by different researchers. Sometimes, researchers only choose external validation datasets that cover all the same measures; other times, researchers choose external validation datasets which overlap with only some measures, in which case the model can only take those measures into account (potentially harming performance). |

**Section C: Confusion Matrix Defining Model Metrics.**

|  |  | **Actual** | |  |
| --- | --- | --- | --- | --- |
|  |  | **Has TRD** | **Does NOT Have TRD** |  |
| **Predicted** | **Predicted to Have TRD** | **True Positive**  Predicted to be treatment-resistant when they are treatment resistant. | **False Positive**  Predicted to be treatment-resistant when they actually will improve with treatment. | **Positive Predictive Value / Precision**  Percent of individuals predicted to be treatment-resistant who truly will not improve with treatment. |
|  | **Predicted to NOT Have TRD** | **False Negative**  Predicted to improve when they actually are treatment resistant. | **True Negative**  Predicted to improve when they truly will improve. | **Negative Predictive Value**  Percent of individuals predicted to improve with treatment who truly will improve with treatment. |
|  |  | **Sensitivity / Recall**  Percent of individuals with treatment resistance who are correctly predicted to be treatment resistant. | **Specificity**  Percent of individuals who will respond to treatment who are correctly predicted to respond to treatment. |  |
