# Supplementary material for: Irremediability in psychiatric euthanasia: examining the objective standard
Source: Psychol Med. 2022 Oct 28;53(12):5729–47. doi: 10.1017/S0033291722002951 (PMC10482705; doi:10.1017/S0033291722002951)
Supplement: Supplementary file 1 [file S0033291722002951sup.zip › S0033291722002951sup002.docx]

**Supplementary Materials 2. List of Abbreviations**

**Psychiatric Diagnoses**

MDD: Major Depressive Disorder

BP: Bipolar disorder

TRD: Treatment-resistant depression

**Diagnostic tools and markers**

EEG: Electroencephalogram

fMRI: Functional Magnetic Resonance Imaging

SPECT: Single-photon emission computerized tomography

MST: Magnetic Seizure Therapy

SNP: Single nucleotide polymorphisms

**Rating Scales**

HAM-D: Hamilton Depression Ration Scale

MADRS: Montgomery-Asberg Depression Rating Scale

CGI: Clinical Global Impression (rating scale)

QIDS: Quick Inventory of Depression Symptomatology

ATHF: Antidepressant Treatment History Form

BDI: Beck Depression Inventory

**Treatment types**

rTMS: repetitive Transcranial Magnetic Stimulation

SSRI: Selective Serotonin Reuptake Inhibitor

AD: Antidepressant

DBS: Deep Brain Stimulation

VNS: Vagus Nerve Stimulation therapy

TCA: Tricyclic antidepressant

**Datasets used**

GSRD: Group for the Study of Resistant Depression

NESDA: Netherlands Study of Depression and Anxiety study

RIS-INT-93: protocol number of a large dataset available via Yoda Project

STAR*D: Sequenced Treatment Alternatives to Relieve Depression Study

TRD-I/III: datasets based on GSRD data

**Methodology**

SVM: Support Vector Machine

PPV: Positive Predictive Value

NVP: Negative Predictive Value

AUC: Area under the ROC curve

Acc.: Accuracy

Bal. Acc.: balanced accuracy

F1: Weighted average of Precision and Recall (Evaluation measure in machine learning)

ROC analysis: Receiver-Operating Characteristic Analysis (Tool for evaluating accuracy of a statistical model**)**
